# Characterization of *Listeria* prophages in lysogenic isolates from foods and food processing environments

**DOI:** 10.1371/journal.pone.0214641

**Published:** 2019-04-01

**Authors:** Hue Thi Kim Vu, Soottawat Benjakul, Kitiya Vongkamjan

**Affiliations:** 1 Department of Food Technology, Prince of Songkla University, Hat Yai, Songkla, Thailand; 2 Department of Veterinary Hygiene and Food Safety, National Institute of Veterinary Research (NIVR), Dong Da, Hanoi, Vietnam; Massey University, NEW ZEALAND

## Abstract

Prophages are commonly found in *Listeria* genomes, potentially enhancing survival or fitness of *Listeria* spp. Currently, there is still limited information on the distribution of prophages among *Listeria* isolates of different allelic types and from various sources. In this study, by using mitomycin C induction, prophages were found in 23/144 isolates (16.0%), including 13 *L*. *monocytogenes* and 10 *Listeria* spp. isolates, resulting in 28 and 11 induced phages, respectively. These prophage-carrying isolates (lysogens) were obtained from foods and food-related environments presenting 3 common allelic types (ATs) of *L*. *monocytogenes* (lineage I, II and IV), 4 ATs of *L*. *innocua* and 1 AT of *L*. *welshimeri*. The likelihood of prophage-carrying isolates of *L*. *monocytogenes* was 14.4 (95% CI: 4.9–35.4), and 18.5 (95% CI: 4.8–50.2) for *Listeria* spp. The 39 induced phages were classified into 3 lysis groups by the host range test against 9 major serotypes of *L*. *monocytogenes* and 5 species of *Listeria*. Most phages were host-specific with higher ability to lyse *L*. *monocytogenes* serotype 4 than other serotypes. The genome size of phages ranged from 35±2 kb to 50±2 kb and belonged to two common phage families, *Myoviridae* and *Siphoviridae*. Restriction analysis classified 19 selected phages into 16 restriction profiles, suggesting highly diverse prophages with at least 16 types. This may contribute to the variation in the genomes of *Listeria*. Information obtained here provides basic knowledge for further study to understand the overall role of prophages in *Listeria*, including roles in survival or fitness in foods and food processing environments.

## Introduction

*Listeria monocytogenes* is an important foodborne pathogen that can cause listeriosis-a serious foodborne illness with mortality rate up to 30% [1]. The genus *Listeria* comprises 17 species. Several new species have been discovered in the past decade, for example, *L*. *marthii* from soil [2]; *L*. *fleischmannii* and *L*. *weihenstephanensis* in cheese and water, respectively [3,4]. Previous studies have reported that various types of foods and food processing environments can be contaminated with *Listeria* spp., including *L*. *monocytogenes* [5,6]. *L*. *monocytogenes* showed good survival in specific environments and they were resistant to the deleterious effects of freezing, drying, and heat [7–9]. There could be some potential factors such as gene transfer, gene gain or loss that may facilitate survival, evolution and speciation of this pathogen.

Bacteriophages (phages) are viruses of bacteria, and are the most common component in the biosphere [10]. Phages can be classified into two groups based on their life cycles [11]. Phages from lytic cycle, called lytic phages, replicate using bacterial machinery and then destroy the host. Phages from lysogenic cycle, called lysogenic phages, have the same lytic capacity but in addition, they can also integrate their DNA into the bacterial chromosome to establish a prophage. The interests of prophage and the host are partly aligned because the lysogen will prolong prophage status [12]. Prophages have several important roles in facilitating the lysogen’s survival [13,14], virulence [15] and phage resistance [16]. Therefore, studying the diversity of prophages and their characteristics (e.g., host specificity) is useful for further studies to understand prophage’s contribution to overall life of the bacteria.

Previous studies have revealed the pervasiveness of prophages in *Listeria* genomes, and multiple prophages were found in a single strain [17,18]. For example, *L*. *innocua* CLIP11262 harbored up to six prophage-like elements, including 5 prophages and 1 monocin [19]. A recent study reported that various types of prophage inserted into the genomes of *L*. *monocytogenes* ST121 [20]. However, these studies mostly have applied bioinformatics analyses to search for prophage regions in *Listeria* genomes. Alternatively, if no sequencing is performed on *Listeria* isolates, induction is an effective approach to examine the presence of prophages. Among the reported inducing agents such as antibiotics, UV radiation, sunlight, temperature, or pressure [21–23], mitomycin C has been reported as the most effective in prophage induction [16,24].

In this study, mitomycin C induction was performed to investigate the distribution of prophage among *Listeria* isolates obtained from various foods and food-processing environments. These isolates were classified based on partial *SigB* sequences, which is useful for the prediction of prophage in each allelic type representing different lineages/ species. Characterization of the induced phages phenotypically and genotypically allows us to better understand prophage diversity, which may contribute to the variation in *Listeria* genomes or to *Listeria* survival and fitness in foods and food-related environments. Moreover, host range data obtained here could allow predicting particular subtypes of *L*. *monocytogenes* or *Listeria* spp. in which gene transfer may occur upon phage infection, leading higher survival of the pathogen in the food production chain.

## Materials and methods

### *L*. *monocytogenes* and *Listeria* spp. isolates used in this study

A total of 144 isolates of *L*. *monocytogenes* and other *Listeria* spp. (non- *monocytogenes*) were used for prophage induction in this study (Table 1). These isolates were previously obtained from multiple sources [5,6,25] and are maintained at the Department of Food Technology, Prince of Songkla University (PSU): animal origin products (n = 38), seafood/ aquatic products (n = 53), vegetable products (n = 8), food contact surfaces (n = 22) and non-food contact surfaces (n = 23). Four *L*. *monocytogenes* reference strains, including F2365, Mack, FSL F2-695 and FSL J1-208 were used as propagating hosts for prophage induction and phage lysate preparation (Table 2). A total of 31 *Listeria* strains/ isolates were used for host range determination. These included 19 reference strains [25,26] obtained from the Food Safety Lab (FSL), Cornell University, and from the Department of Medical Science Thailand (DMST), Ministry of Health, Thailand. Additional 12 isolates of *L*. *monocytogenes* and *Listeria* spp. were selected from the collection of 144 isolates used in this study [5,6,25]. Of these, eight *L*. *monocytogenes* hosts were subjected to serotype classification by multiplex PCR following the protocol of Doumith et al. [27] combined with the classification of *sigB* allelic types.

**Table 1:** Source of *L*. *monocytogenes* and *Listeria* spp. isolates used in this study.

Source of isolates	Number of isolates		
	*L*. *monocytogenes*	*Listeria* spp.	Total
Animal origin products (Ani)	18	20	38
Seafood/ aquatic products (Sea)	27	26	53
Vegetable products (Veg)	3	5	8
Food contact surfaces (FCS)	19	3	22
Non-food contact surfaces (NFCS)	23	0	23
Total	90	54	144

**Table 2:** *L*. *monocytogenes* and *Listeria* spp. strains/ isolates subjected to mitomycin C induction and host range determination.

Strain/ isolate ID	Lineage/ species	Serotype	Source
*L*. *monocytogenes*^a^ and other *Listeria* spp. reference strains			
FSL J1-175	I	1/2b	Water
FSL J1-194	I	1/2b	Human
FSL J1-169	I	3b	Human
FSL J1-049	I	3c	Human
FSL R2-574 (F2365)*	I	4b	Food
FSL F6-367(Mack)*	II	1/2a	Lab strain
FSL R2-0559	II	1/2a	Food
FSL J1-094	II	1/2c	Human
FSL C1-115	II	3a	Human
FSL F2-695*	IIIA	4a	Human
FSL F2-501	IIIA	4b	Human
FSL J2-071	IIIA	4c	Animal
FSLW1-110	IIIC	4b	Unknown
FSL J1-208*	IV	4a	Animal
FSL J1-158	IV	4b	Animal
DMST-9011	*L*. *innocua*		Unknown
DMST-9012	*L*. *ivanovii*		Unknown
FSL C7-0084	*L*. *marthii*		Soil, forest
FSL C7-0015	*L*. *seeligeri*		Soil
*L*. *monocytogenes* and other *Listeria* spp. isolates			
PSU-KV-032LM	I	1/2b, 3b	Environmental-FCS
PSU-KV-042LM	I	1/2b, 3b	Environmental-NFCS
PSU-KV-038LM	I	4b	Environmental-FCS
PSU-KV-105LM	I	4b	Seafood/aquatic product
PSU-KV-148LM	II	1/2c	Animal origin product
PSU-KV-159LM	II	1/2c	Animal origin product
PSU-KV-108LM	IV	4a, 4c	Seafood/aquatic product
PSU-KV-120LM	IV	4a, 4c	Animal origin product
PSU-KV-114LS	*L*. *innocua*		Animal origin product
PSU-KV-146LS	*L*. *innocua*		Animal origin product
PSU-KV-131LS	*L*. *welshimeri*		Seafood/aquatic product
PSU-KV-181LS	*L*. *welshimeri*		Vegetable product

^a^Four reference strains with ‘*’ were used as the propagating hosts for mitomycin C induction and phage lysate preparation.

### *SigB* allelic typing

Typing of a partial *sigB* gene has been used for allelic type classification and species confirmation of *Listeria* isolates in previous studies [5,28,29]. Of the 144 *Listeria* isolates used for prophage induction in this study, 60 have been assigned an allelic type (AT) previously [5,6]. *SigB* allelic typing was performed to 84 *Listeria* isolates in this study. The protocol of Nightingale et al. [28] was followed for PCR amplification of a partial *sigB* gene (780 bp) using the same primers. The purified PCR products were sequenced at Macrogen Inc. (Seoul, South Korea). Allelic type classification was performed using the *SigB* allelic type database of all species of *Listeria* (kindly provided by Prof. Martin Wiedmann and Dr. Renato H Orsi, Cornell University, Ithaca, New York). A phylogenetic tree was generated by MEGA5 program [30] using sequences of the allelic types found in this study with some closely related allelic types in the database mentioned above. The maximum likelihood method with gamma distribution was used for constructing the tree with 1,000 bootstrap replications [29,31].

### Induction of *Listeria* prophage by mitomycin C and phage lysate preparation

The culture of an isolate was prepared by inoculating an isolated colony in 5 ml of Luria Bertani (LB) broth (Oxoid, UK) supplemented with 50 mM morpholinepropanesulfonic acid (MOPS), 1% (wt/vol) glucose, 10 mM CaCl_2_, and 10 mM MgCl_2_ (LB-MOPS-Glu-salts) [26]. The culture was incubated at 30°C (220 rpm) to reach an optical density (at 600 nm) of 0.4 to 0.5, and a 1 ml-aliquot was mixed with mitomycin C (Sigma-Aldrich, St Louis, USA) to a final concentration of 1 μg/ml (modified from [32]). The mixture was subsequently incubated for 7 h. Then, 200 μl of this mixture was later mixed with 100 μl of a given propagating host in a total volume of 2 ml of LB MOPS, followed by an incubation for 18 h at 30°C (220 rpm). The double layer technique was applied with the filtered lysate of the overnight co-culture following the procedure described by Vongkamjan et al. [26]. Plaque formation was observed as appearance of induced phage. An isolated plaque representing a distinct plaque morphology type (A: translucent plaque, rather round shape, Φ ≥ 1 mm; B: turbid at the edge, star-shape, Φ = 0.5–1 mm; C: turbid zone, Φ ≤ 0.5 mm; D: clear zone, round, Φ = 1 mm; E: turbid zone, tiny, Φ ≤ 0.2 mm) was selected for three-time-purification, followed by phage lysate preparation by the double layer method [26]. Titers of induced phages were determined by spotting 5μl of ten-fold serial dilutions of phage on the propagating host lawn. High titer of phage lysate was kept at 4°C for further analysis. The likelihood of prophage-carrying isolates among *L*. *monocytogenes* and *Listeria* spp. was determined as odd ratio with 95% confidence interval (95% CI) using a generalized linear model in R program version 3.1.2 (https://cran.r-project.org).

### Host range determination of the induced phages

Phage host range determination was performed by spotting 5 μl of diluted phage representing 100×RTD (routine test dilution) [26], approximately 10^6^–10^7^ PFU/ml, on the 31 *Listeria* hosts mentioned above. Each spot on the lawn was examined and recorded for lysing (+) or no lysing (-) after overnight incubation at 30°C [26]. The experiment was carried out in triplicate. Then, a clustering analysis based on lysis ability of the induced phages against the tested hosts was performed using R program.

### Estimation of phage genome size

Pulsed-Field Gel Electrophoresis (PFGE) analysis was used to estimate genome size of the phages as described previously [25,26,33]. Briefly, high-titer lysate of a given phage (10^7^–10^9^ PFU/ml) was used to prepare a plug with 1.3% low-melting-point agarose. PFGE analysis was performed on a CHEF-DR III system (Bio-Rad, Hercules, CA, USA) in 0.5×TBE buffer (1 M Tris, 0.5M EDTA and boric acid, pH 8.0) for 22 h with a 0.5 s to 5.0 s switch time, 6 V/cm, and an included angel of 120° [26]. Genome estimation was performed using Uvitec UVI-1D software (Uvitec Limited Co., Cambridge, UK) with the tool for molecular weight estimation.

### Restriction enzyme analysis

A total of 19 phages representing each genome size group, but from different lysis groups and sources were selected for restriction enzyme analysis. DNA of the induced phages was extracted by phenol/chloroform as described previously [26]. Restriction analysis was performed using Fast digest HindIII (Thermo Fisher Scientific, MA, USA) and Eco*R*I (Vivantis, Selangor Darul Ehsan, Malaysia), following the manufacturers’ instructions. Two restriction profiles were considered different when at least one distinguishing band was present [32].

### Transmission electron microscopy (TEM)

Examination of the phage morphology and family was performed with four induced phages selected to represent a given genome size group. A 3 μl-drop of freshly prepared lysate of a given phage (10^8^ PFU/ml) was deposited onto carbon-coated copper grid. The grid was left to dry for 15 s, then slowly stained with 20 μl of uranyl acetate (2%, pH 4.5) [32,34]. The imaging was done at 160 kV with a transmission electron microscope JEM-2010, JEOL (Japan) at the Scientific Equipment Center, Prince of Songkla University, Hat Yai, Thailand.

## Results

### Classification of allelic types based on *Listeria* partial *sigB* sequences

**Fig 1:**
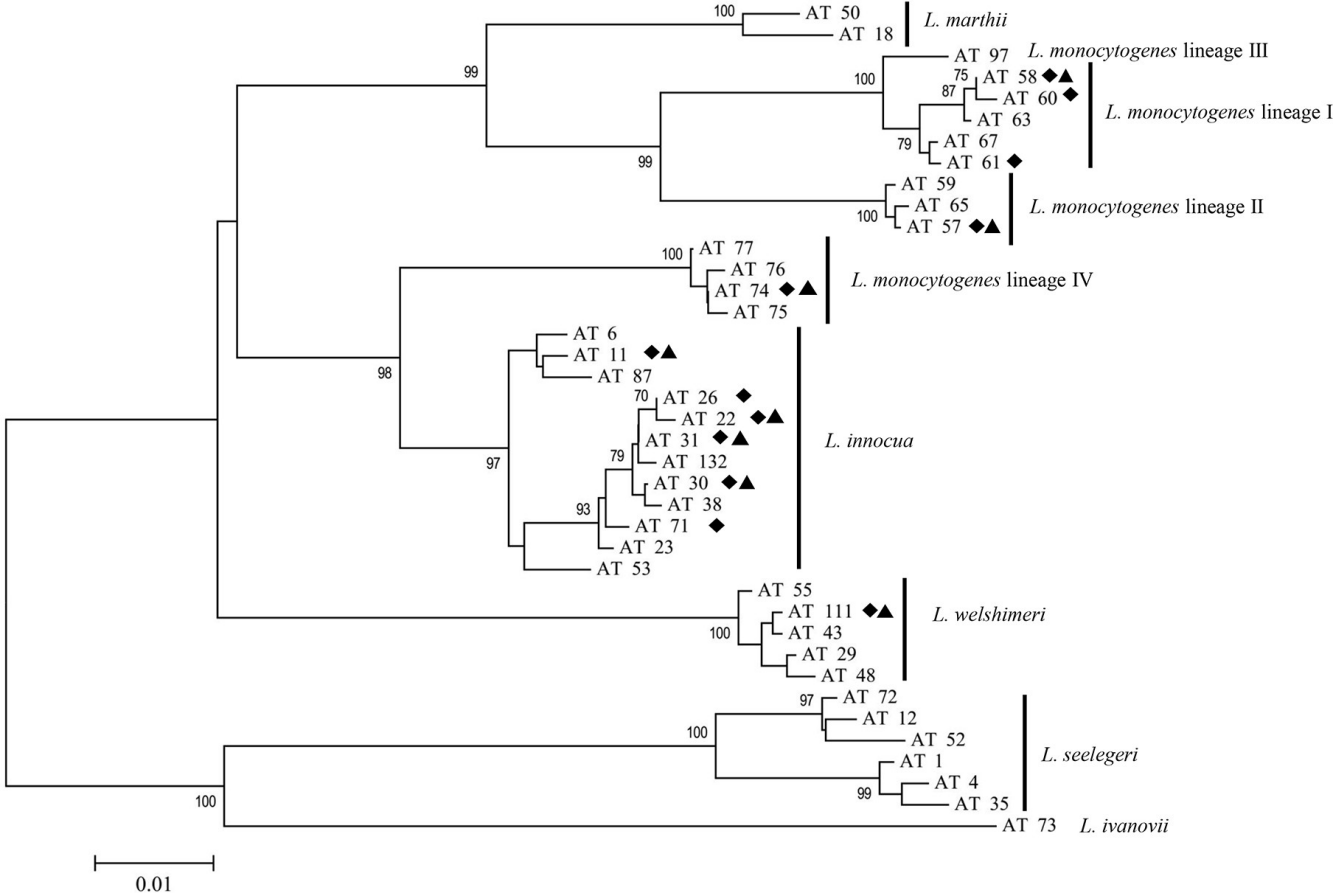
Neighbor-joining tree describes the *sigB* allelic type (AT) of 144 isolates included in this study (♦) and some closely related allelic types from the database. The maximum likelihood method and a gamma distribution were used to construct the tree with 1,000 bootstrap replications. Only bootstrap values ≥70% are presented on the tree. Triangles (▲) indicates that the allelic type contained prophage.

Overall, a total of 144 *L*. *monocytogenes* and *Listeria* spp. isolates were subjected to partial *sigB* sequencing analysis to classify them into allelic types (Table 3 and Fig 1). Classification of these isolates showed 12 allelic types, which represented lineages I, II or IV of *L*. *monocytogenes*, or other species, including *L*. *innocua* and *L*. *welshimeri*. In the 90 *L*. *monocytogenes* isolates used in this study, five allelic types were observed. Of which, AT 58, AT 60 and AT 74 were commonly found in the majority of isolates. Allelic types 58 and 60 represented *L*. *monocytogenes* lineage I while AT 74 represented *L*. *monocytogenes* lineage IV. In the 54 *Listeria* spp. isolates, AT 111 and AT 30 were common allelic types found in this study; these isolates could be classified as *L*. *welshimeri* and *L*. *innocua*, respectively.

**Table 3:** Presence of prophages in the isolates of *L*. *monocytogenes* and *Listeria* spp.

Lineage/ species		*SigB* allelic type (AT)	No. of isolates	No. of lysogens	The likelihood of prophage-carrying isolates (95% Confidence interval)
***L*. *monocytogenes***					**14.4 (4.9–35.4)**
	Lineage I (n = 60)	AT 58	24	8	
		AT 60	22	-	
		AT 61	14	-	
	Lineage II (n = 4)	AT 57	4	4	
	Lineage IV (n = 26)	AT 74	26	1	
***Listeria* spp.**					**18.5 (4.8–50.2)**
	*L*. *innocua* (n = 27)	AT 11	6	1	
		AT 22	3	1	
		AT 26	1	-	
		AT 30	13	2	
		AT 31	3	2	
		AT 71	1	-	
	*L*. *welshimeri* (n = 27)	AT 111	27	4	

### Distribution of prophages in *Listeria* isolates obtained from foods and food-related environments

Mitomycin C could induce the prophages in 23/144 (16.0%) of *L*. *monocytogenes* and *Listeria* spp. isolates (Table 3). These prophage-carrying isolates belonged to 8 out of 12 allelic types found among the tested isolates (Table 3 and Fig 1). The eight common allelic types associated with the occurrence of prophages included 3 ATs of *L*. *monocytogenes* [AT 58 (lineage I), AT 57 (lineage II), and AT 74 (lineage IV)] and 5 ATs of *Listeria* spp. [AT 11, AT 22, AT30, and AT 31 (*L*. *innocua)*; and AT 111 (*L*. *welshimeri)*]. Overall, the likelihood of prophage-carrying isolates of *L*. *monocytogenes* was 14.4 (95% CI: 4.9–35.4) and 18.5 (95% CI: 4.8–50.2) for *Listeria* spp.

Based on the presence of distinct types of plaque morphology based on the size, shape and the turbidity of each examined plaque, 39 inducible phages were obtained from 23 lysogenic isolates (Table 4). The majority of phages (23 phages) were obtained from eight lysogens of *L*. *monocytogenes* lineage I. Other four phages were from four lysogens of lineage II and only one phage was from lysogens of lineage IV. Ten lysogens of *Listeria* spp. yielded 11 phages, including six phages from *L*. *innocua* and five phages from *L*. *welshimeri*. Among four *L*. *monocytogenes* hosts used as propagating hosts, two *L*. *monocytogenes* strains of serotype 4a were common hosts for phage propagation, while the strain FSL F2-695 was propagating host for phages from the lysogens of *L*. *monocytogenes* lineage I and *L*. *innocua*. The strain FSL J1-208 was suitable as the propagating host for phages from the lysogens of *L*. *monocytogenes* lineage I and *L*. *welshimeri*. The strain *L*. *monocytogenes* serotype 1/2a (Mack) was a suitable host for phages from the lysogens of *L*. *monocytogenes* lineage II. F2365 (serotype 4b) could be propagating host for phages from the lysogens of *L*. *monocytogenes* lineage I and IV. While one lysogen of lineage IV could yield a phage on only F2365 host, some lysogens such as PSU-KV-165LM and PSU-KV-167LM provided up to five phages in three propagating hosts.

**Table 4:** Distribution of induced *Listeria* phages on different propagating hosts.

Lineage/ species	Lysogen ID^a^	Allelic type (AT) of lysogen	Induced phages ID^b^ on each propagating host (genome size group)			
			Mack (1/2a)	FSL F2-695 (4a)	FSL J1-208 (4a)	F2365 (4b)
*L*. *monocytogenes* lineage I						
	112LM	AT 58	-	**LP013** (4)	LP012 (1)	**LP010**/011 (1)
	133LM	AT 58	-	-	**LP017** (2)	LP016 (2)
	134LM	AT 58	-	-	LP019 (2)	**LP018** (2)
	160LM	AT 58	-	-	LP027/028 (1)	-
	165LM	AT 58	-	**LP034** (3)	LP032/**033** (1)	LP030/031 (1)
	167LM	AT 58	-	LP039 (4)	LP037/038 (1)	**LP035**/036 (1)
	036LM	AT 58	-	**LP041 (4)**	-	**LP040** (2)
	038LM	AT 58	-	-	-	LP042 (2)
*L*. *monocytogenes* lineage II						
	148LM	AT 57	LP022 (2)	-	-	-
	149LM	AT 57	**LP023** (2)	-	-	-
	150LM	AT 57	LP024 (2)	-	-	-
	159LM	AT 57	LP026 (2)	-	-	-
*L*. *monocytogenes* lineage IV						
	218LM	AT 74	-	-	-	**LP047** (1)
*L*. *innocua*						
	143LS	AT 31	-	LP020 (3)	-	-
	145LS	AT 31	-	**LP021** (3)	-	-
	152LS	AT 30	-	LP025 (3)	-	-
	199LS	AT 30	-	**LP045** (2)	-	-
	192LS	AT 11	-	**LP044** (4)	-	-
	200LS	AT 22	-	**LP046** (4)	-	-
*L*. *welshimeri*						
	104LS	AT 111	-	-	**LP009** (2)	-
	130LS	AT 111	**LP014**/015 (2)	-	-	-
	164LS	AT 111	-	-	**LP029** (2)	-
	181LS	AT 111	-	-	**LP043** (2)	-

^a^ID of *Listeria* lysogen has a prefix of “PSU-KV-”.

^b^ID of induced *Listeria* phages has a prefix of “PSU-VKH-”. Multiple induced phages listed are separated by “/”. Phages in bold indicates that were selected for restriction enzyme analysis.

### Lysis ability of the induced phages against 31 *Listeria* hosts

**Fig 2:**
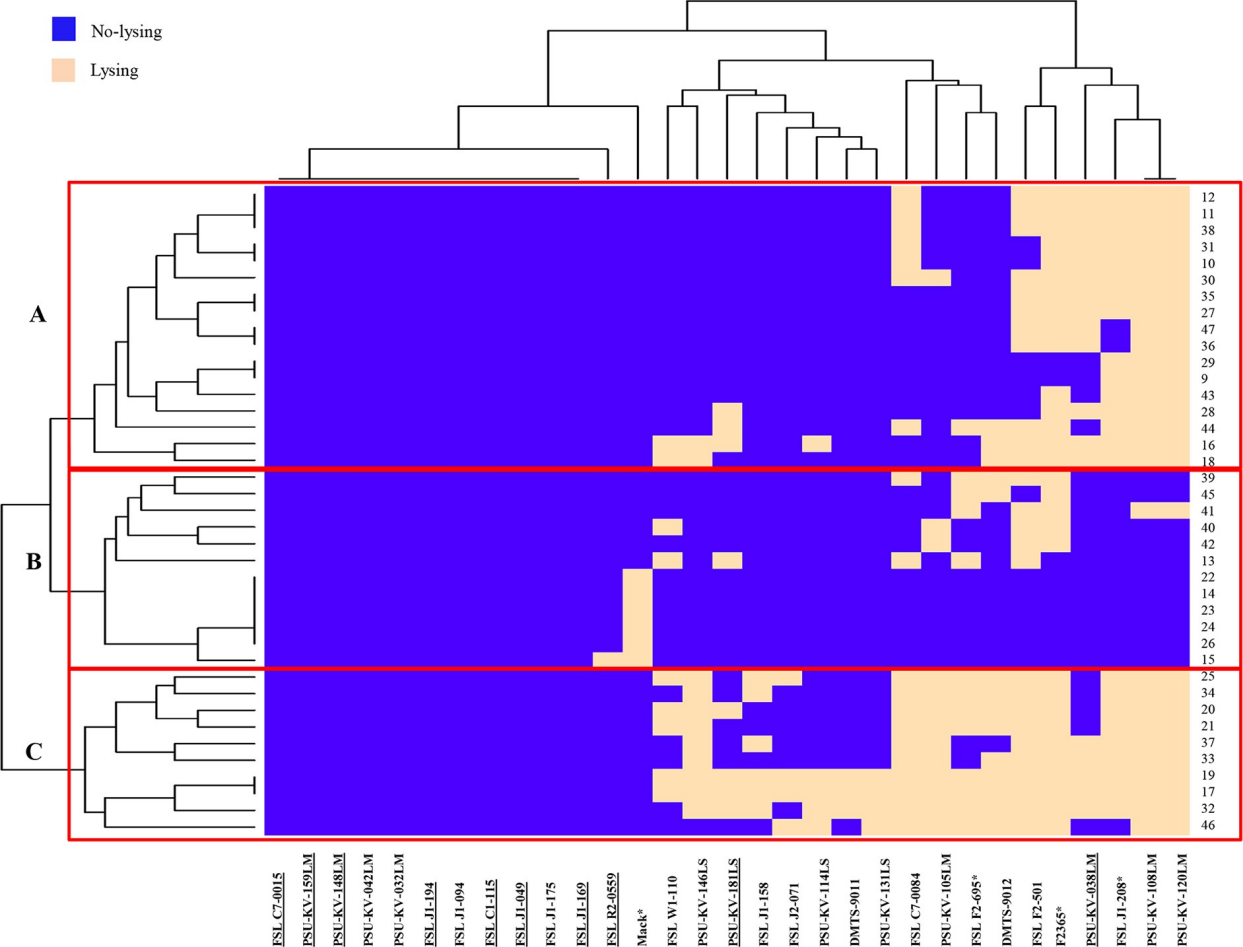
Clustering analysis shows lysis capabilities of 39 induced *Listeria* phages against 31 tested hosts. Blue represents no-lysing and beige represents lysing of a given host. Host strains are shown on the x-axis, while induced phages are shown in the y-axis. Clusters of the induced phages are designated A to C based on similarities of the lysis profiles on 31 hosts. The clustering was done using R-program v.3.1.2. Underlined hosts represent lysogenic hosts.

The host range of 39 induced phages was evaluated on 19 *Listeria* reference strains and 12 *Listeria* isolates from the collection of those isolates used in this study. These hosts were selected to represent 9 major *L*. *monocytogenes* serotypes and 5 distinct *Listeria* species. Clustering analysis based on the lysis similarity classified these 39 phages into 28 lysis profiles, presenting 3 major lysis groups (A, B and C) (Fig 2). Each lysis group included 10–17 induced phages. Group B contained those phages (n = 12) that were host-specific with the ability to lyse only 1–5 host strains (<16%) of *L*. *monocytogenes* serotype 4 and *L*. *marthii*. Phages (n = 17) in group A showed similar host range as those in group B (as host-specific phages), however, they could also lyse Mack (1/2a) and *L*. *welshimeri*. In comparison, phages (n = 10) in group C had a broader host range than those in groups A and B. These phages could lyse 10–18 hosts (32–58%) of *L*. *monocytogenes* serotype 4 and other *Listeria* species used as hosts, except *L*. *seeligeri*. Overall, the majority of phages (30/39 phages) could lyse hosts of *L*. *monocytogenes* serotype 4. Interestingly, 10/31 hosts resistant to the induced phages were lysogens.

### Estimated genome size of the induced phages

Genome estimation of the induced phages by PFGE classified them into four groups (Table 4), including group 1 (35±2 kb, 14 phages), group 2 (40±2 kb, 16 phages), group 3 (45±2 kb, 4 phages), and group 4 (50±2 kb, 5 phages). Most phages were in groups 1 and 2. Group 1 contained phages induced from lysogens of *L*. *monocytogenes* lineage I and the only lysogen of *L*. *monocytogenes* lineage IV, while group 2 contained phages induced from lysogens of all *L*. *monocytogenes* lineages and *Listeria* species found in this study (except *L*. *monocytogenes* lineage IV). In comparison, groups 3 and 4 included phages induced from lysogens of *L*. *monocytogenes* lineage I and *L*. *innocua*. Interestingly, many *Listeria* lysogens harbored different induced phages with different genome size representing different prophage types in their genomes.

### Restriction analysis of the induced phages

**Fig 3:**
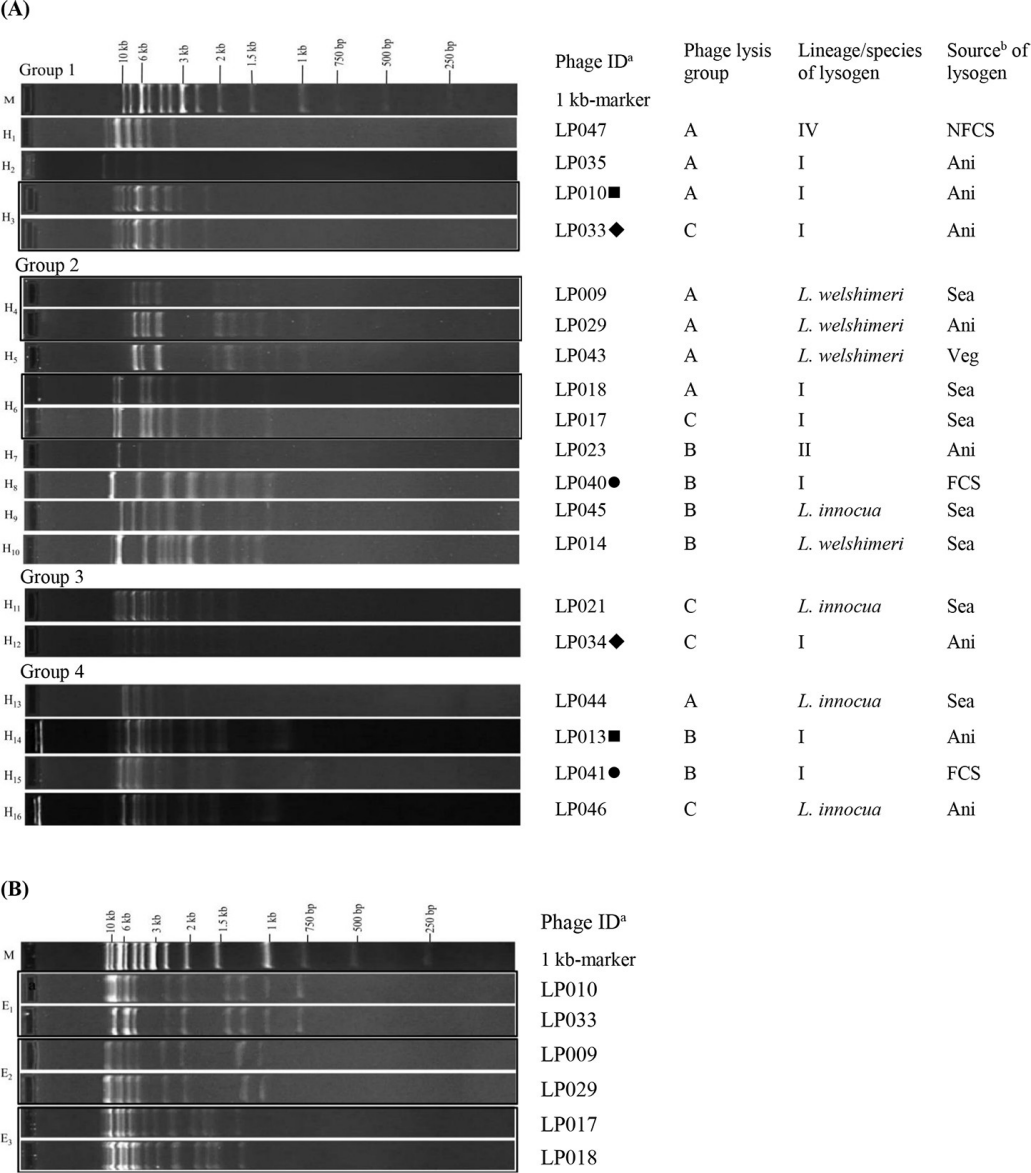
Restriction analysis of induced *Listeria* phages with enzymes HindIII and Eco*R*I. HindIII-restriction profiles of selected induced phages grouping by genome size (group 1 to 4) (Fig 3A). Eco*R*I-restriction profiles of induced phages that showed similar restriction profiles by HindIII (Fig 3B). ^a^“M” is a 1-kb molecular maker. Induced phages obtained from a single lysogen are marked with the same symbol next to the phage ID. Phages within a box had the same restriction pattern. ^b^Refer to Table 1 for abbreviations of the lysogen sources.

Restriction enzyme analysis using HindIII was performed with 19 representative phages, resulting in 16 restriction profiles (H_1_ to H_16_) (Fig 3). In each genome size group, 2–7 different restriction profiles were obtained. Seven different profiles were obtained in phage genome size group 2 (40±2 kb). The profiles H_3_ (group 1) and H_6_ (group 2) were both observed in two phages from the lysogens of *L*. *monocytogenes* lineage I obtained from the same source. For group 2, profile H_4_ was found in two phages (LP009 and LP029) from the lysogens of *L*. *welshimeri* obtained from different sources. Two and four distinct restriction profiles were observed in phages of genome groups 3 and 4, respectively.

Phages with the same HindIII restriction profiles (H_3_, H_4_, or H_6_) were further analyzed by enzyme Eco*R*I. Results showed that two phages with identical HindIII profiles had identical Eco*R*I profiles (E_1_, E_2_ or E_3_). In summary, by using two restriction enzymes, these 19 phages could be classified into 16 restriction profiles, suggesting 16 different prophage types. Interestingly, up to seven prophage types were obtained from the lysogens of *L*. *monocytogenes* lineage I (AT 58). In addition, three and four prophage types were obtained from the lysogens of *L*. *welshimeri* (AT 111) and *L*. *innocua* (AT 11, AT 22, AT 30, AT 31), respectively.

### TEM analysis of the induced phages

**Fig 4:**
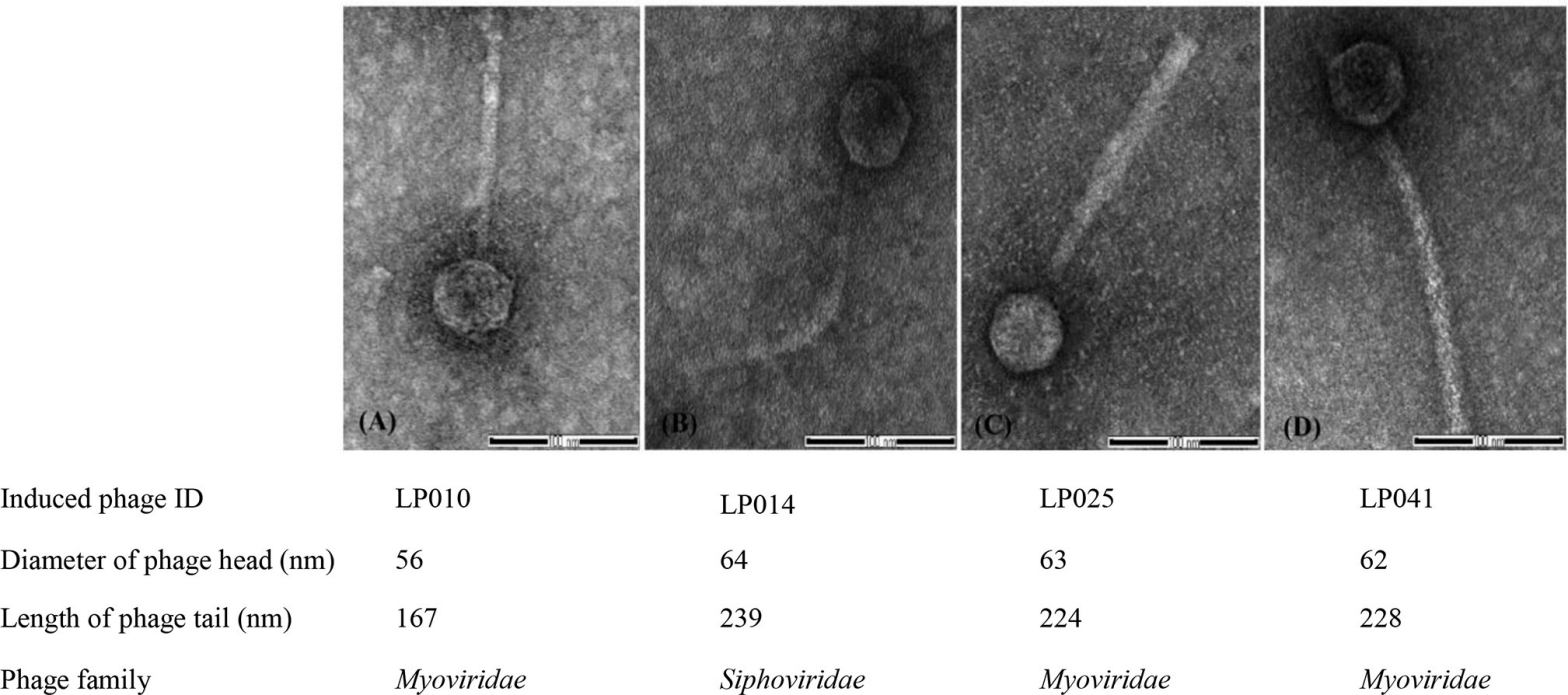
Morphology of induced *Listeria* phages by TEM analysis. Induced phages were stained with 2% uranyl acetate (pH 4.5) and visualized at a final magnification of 100kx. All panels are shown at the same scale with the scale bar indicating 100 nm.

Morphology of four phages selected to represent the four genome size groups was examined by TEM (Fig 4). Morphology observation showed that three phages belonged to the *Myoviridae* family with an isometric head (diameter 56 to 63 nm) with long contractile tail (167 to 228 nm) with a sheath. These phages were from the lysogens of *L*. *monocytogenes* lineage I or *L*. *innocua* with the genome sizes 35±2 kb, 45±2 kb and 50±2 kb. Another phage was classified to the *Siphoviridae* family with a hexagonal head (diameter of 64 nm) and longer non-contractile tail (239 nm). This phage was from the lysogen of *L*. *welshimeri* and had genome size 40±2 kb.

## Discussion

### Distribution of prophages among *L*. *monocytogenes*, *L*. *innocua*, and *L*. *welshimeri* obtained from foods and food-related environments

A total of 39 induced phages were obtained from the 23/144 isolates of *L*. *monocytogenes* and *Listeria* spp. tested (16%). Prophages were detected in most allelic types found in this study (8/12) of *L*. *monocytogenes* lineage I, II and IV, *L*. *innocua*, and *L*. *welshimeri*. No isolates of *L*. *monocytogenes* in the tested collection belonged to lineage III, therefore the results lack information on the prophage distribution from isolates of this lineage.

Prophages were found to be absent or rare in five specific allelic types representing *L*. *monocytogenes* lineages I, IV and *L*. *innocua*. There is still limited information to explain why those allelic types are lack of presence of prophage. However, we speculate that the isolates of these lineages are particularly resistant to uptake of extraneous DNA as suggested previously [35]. Another possible reason could be the lack of prophage insertion sites for these lineages as opposed to *L*. *monocytogenes* lineage I and II [17,18]. Interestingly, finding here may link to the host range data; *L*. *monocytogenes* serotype 1/2 and 3 (mostly belonged to lineages I, II) are resistant to phages as those hosts could be lysogens containing prophage sequences that are homologous to the induced phages [36]. Another possibility could be appropriated propagating host in the induction as mentioned in previous studies [14,37]. This hypothesis was supported when different sets of prophages were obtained using different propagating hosts, even if those hosts represented serotype 4a (FSL F2-695 and FSL J1-208).

In addition, our study showed the likelihood of having prophages in *L*. *monocytogenes* isolates as 14.4 (95% CI: 4.9–35.4) and 18.5 (95% CI: 4.8–50.2) for the isolates of *Listeria* spp. (including *L*. *innocua* and *L*. *welshimeri*). It seems reasonable as *L*. *innocua* and *L*. *welshimeri* were reported that derived from *L*. *monocytogenes* through early evolutionary events of gene acquisition by phage transduction [38]. Therefore, isolates of these *Listeria* species may have more insertion site for prophages to incorporate into the host chromosomes. In addition, in this study, different prophage types were found among *Listeria* lysogens or even within a single lysogen. Similarly, genome analysis has previously revealed multiple prophages in *Listeria* genomes, especially in *L*. *monocytogenes* and *L*. *innocua* [17–19]

### Induced *Listeria* phages appear to be host-specific with higher ability to lyse *L*. *monocytogenes* serotype 4 than other serotypes

Induced *Listeria* phages in this study showed different lysis profiles with 74% of these represented host-specific phages. A previous study revealed that lysogenic *Listeria* phages have narrower host range than isolated *Listeria* phages [39]. The majority of *Listeria* phages isolated from a turkey processing plant showed broad host ranges [40]. In related bacteria, phages obtained from lysogenic strains of *Streptococcus iniae* were reported to have narrow lytic spectrum [41]. This can be explained by the unique characteristic of induced phages as they have the ability to incorporate their genome in the host chromosome instead of lysing the host.

In this study, most induced phages could lyse the hosts of *L*. *monocytogenes* serotype 4. Similarly, *Listeria* phages from silage or turkey processing environments were highly susceptible to *L*. *monocytogenes* serotype 4 strains [26,40]. Moreover, the induced phages could not lyse *L*. *monocytogenes* serotype 1/2 and serotype 3 hosts. This is of interest since serotype 1/2a is linked to the increasing cases of listeriosis in the last decade [42,43]. Therefore, we speculated that prophages may facilitate the survival of *Listeria* hosts. Another potential support is that the differences in phage susceptibility between serotypes of *L*. *monocytogenes* can be explained by the different in structure of cell wall teichoic acids (WTA). This is because *L*. *monocytogenes* serotype 4 contains WTA with terminal glucose and galactose residues, which is important for phage adsorption [44,45] and further facilitation for phages to lyse the host.

Most *Listeria* species used as hosts for the host range determination were sensitive to the induced phages, except *L*. *seeligeri*. This may be because the *L*. *seeligeri* strain is a lysogen. We also observed that the induced phages were likely to be resistant to 10/31 lysogenic *Listeria* hosts. This supports the hypothesis that lysogens could have phage resistance in certain bacterial hosts, because the bacteria may harbor specific (pro)phage sequences that could increase their survival without being affected by phage with homologous sequence as reported in *Oenococcus oeni* [36]. However, sequencing analysis of the induced phage and its host is still needed to elucidate the phage resistance characteristics.

### Induced phages show highly similar genome size as previously reported temperate *Listeria* phages, but rather high genetic diversity and belong to two common phage families

The induced phages in this study showed genome size ranged from 35±2 kb to 50±2 kb and were classified into four groups. Similarly, genomes of previously reported temperate *Listeria* phages also had sizes from 35 kb to 48 kb [46–48]. Temperate phages typically have smaller genomes, that may contain only the necessary sequences for their replication and encapsulation [49,50]. Only the basic, important genes coding for six main functional modules were present in the genomes of temperate phages [47,51]. However, genomes of lytic phage contained a number of additional gene coding sequences with no function [34].

Restriction analysis of 19 selected phages using enzymes HindIII and Eco*R*I resulted in 16 restriction profiles, suggesting considerable diversity of prophages in the genome of *Listeria* lysogens. The diversity of prophages may contribute to the variation of host genomes by prophage incorporation as mentioned in a previous study [18]. Another study has also shown that the differences in a 42-kb-prophage sequence could differentiate four examined *L*. *monocytogenes* strains [52]. In this current study, seven and three prophage types were found in a single allelic type (AT 58 and AT 111, respectively). This suggests the usefulness of prophage to the classification of *Listeria* isolates of the same allelic types.

Two common families *Myoviridae* and *Siphoviridae* were observed among the induced phages in this study. LP014 belonged to *Siphoviridae* family with long, non-contractile tails, which is the most common among phages (60%) [53]. The morphological characteristics revealed that our *Siphoviridae* phage was similar to the *Listeria* phages LP-032-2 and LP-032-3 with a head diameter of 53–55 nm and a tail length of 160–297 nm reported previously [34].

In summary, this is the first study that investigated the distribution of phages induced from *Listeria* isolates of different allelic types of distinct *L*. *monocytogenes* lineages or *Listeria* species. Characterization of induced phages allows us to better understand their diversity and lysis ability against *Listeria* hosts representing different *L*. *monocytogenes* serotypes and distinct *Listeria* species. Diversity of prophage may have contributed to the genetic diversity of *Listeria* spp. isolated from foods and food-related environments in Thailand. Recombination and mosaicism caused by prophages in *Listeria* genomes may occur and gene transfer may be affected and could later drive host survival and fitness in foods or food-associated environments.
